# Cytokine-Driven Immune Phenotypes at Delivery as Indicators of Malaria Infection Among Primigravidae in Burkina Faso: An Exploratory Analysis

**DOI:** 10.3390/tropicalmed11030080

**Published:** 2026-03-12

**Authors:** Ousmane Traore, Toussaint Rouamba, Serge Henri Zango, Hermann Sorgho, Innocent Valea, Maminata Traore-Coulibaly, Henk D. F. H. Schallig, Halidou Tinto

**Affiliations:** 1 Institut de Recherche en Sciences de la Santé, Unité de Recherche Clinique de Nanoro (IRSS-URCN), Nanoro BP 18, Koudougou 40400, Burkina Faso; rouambatoussaint@gmail.com (T.R.); henrizango@yahoo.fr (S.H.Z.); hsorgho@hotmail.com (H.S.); innocentvalea@crun.bf (I.V.); traore_maminata@yahoo.fr (M.T.-C.); halidoutinto@gmail.com (H.T.); 2 Laboratory for Experimental Parasitology, Department of Medical Microbiology, and Infection Prevention, Amsterdam University Medical Center, 1105 AZ Amsterdam, The Netherlands; h.d.schallig@amsterdamumc.nl; 3 Amsterdam Institute for Global Health and Development, 1105 BP Amsterdam, The Netherlands

**Keywords:** malaria, primigravidae, immune biomarkers, TNF-α, IL-6

## Abstract

In malaria-endemic regions, women remain vulnerable to *Plasmodium falciparum* infection at the time of delivery. However, the immunological mechanisms underlying infection-associated inflammation in primigravid women remain poorly characterized. This exploratory study investigated cytokine-based immune profiles reflecting malaria infection status at delivery. We assessed 33 primigravid women from Nanoro, Burkina Faso (mean age 19 years; range 18–20.5) at childbirth. Antibody responses to *P. falciparum* antigens (*Pf*CSP, *Pf*AMA-1, and EBA-175) and plasma levels of cytokines (IL-4, IL-10, IL-6, TNF-α, and IFN-γ) were quantified using enzyme immunoassays. Multivariate analyses, including principal component analysis (PCA) and hierarchical clustering, identified three distinct immune profiles: (1) a low-inflammatory cluster with reduced IL-6 and TNF-α, (2) a TNF-α–dominant cluster, and (3) a highly pro-inflammatory cluster with elevated IL-6 and TNF-α. Cluster stability was supported by bootstrap analysis (AU ≥ 92%). All women in the most inflammatory cluster were *P. falciparum*–positive at delivery (Fisher’s exact test, *p* = 0.04; exploratory association). These cytokine-driven profiles reflect biologically distinct inflammatory states associated with concurrent infection at delivery rather than predictive immune predispositions. The findings underscore the potential of cytokine profiling as a hypothesis-generating tool to guide future longitudinal studies on immune regulation and the postpartum period.

## 1. Introduction

Malaria remains a pressing global health concern, particularly in regions such as sub-Saharan Africa where the disease is endemic. In 2022, the World Health Organization reported approximately 249 million malaria cases and over 600,000 associated deaths, with pregnant women and children under five being the most affected [1]. Among pregnant women, primigravidae, those experiencing their first pregnancy, face increased susceptibility to malaria and its complications due to limited exposure and immunological naivety [2,3]. While malaria risk during pregnancy is well-documented, evidence suggests that vulnerability may persist into the early postpartum period, reflecting incomplete immune recovery or residual placental parasitaemia [3,4,5,6]. Primigravidae may enter the postpartum period with an immunological alteration that has been associated with malaria in previous studies [7,8,9]. Elevated levels of interleukin-10 (IL-10) and soluble TNF receptor II (sTNF-RII) have shown promise as diagnostic biomarkers in primigravid women, with strong correlations to parasite density [10]. Additionally, iron biomarkers such as ferritin and soluble transferrin receptor are linked to increased malaria risk, suggesting iron status plays a modulatory role in susceptibility [11]. In recent years, immunoprofiling has emerged as a powerful tool to identify host susceptibility to infection by characterizing individual immune signatures. Studies have shown that cytokine balance and antibody responses can stratify individuals based on clinical risk, even in the absence of active infection [12,13]. Additionally, system biology approaches have demonstrated the potential utility of such immune profiling for characterizing disease-associated immune patterns for disease outcomes [14]. This study aligns with ongoing global efforts to harmonize immunological and computational frameworks for infection surveillance and computational modeling. Recent advances in translational research have demonstrated that integrative immunoprofiling can substantially improve disease prediction and patient stratification across infectious syndromes [15]. Furthermore, computational biology frameworks and network-based models now enable high-dimensional clustering of immune biomarkers for scalable, reproducible data integration across studies [16]. These global collaborative efforts, including vaccine and immunology network harmonization initiatives [17] and open-access digital platforms for infectious disease modeling [18], provide a robust framework for contextualizing cytokine-driven biomarker research in malaria-endemic populations.

In this study, we set out to explore whether immune profiles at the time of delivery could help reveal distinct patterns among first-time mothers and help reveal distinct immune patterns at delivery and their association with concurrent malaria infection. Given the scarcity of data on the immunological state of women at the time of delivery, particularly in first pregnancies, this study aimed to generate exploratory evidence rather than confirmatory conclusions. We therefore used a data-driven multivariate approach to identify cytokine- and antibody-based immune profiles that might signal increased infection risk at delivery and generate hypotheses for future longitudinal research. Rather than focusing on any single marker, we looked at a combination of antibody responses to *Plasmodium falciparum* antigens (*Pf*CSP, *Pf*AMA-1, EBA-175) alongside several key cytokines (IL-4, IL-10, IL-6, TNF-α, and IFN-γ). Using principal component analysis and hierarchical clustering, our goal was to uncover broader immune signatures and understand how they might relate to malaria status at delivery [19,20].

The cytokines analyzed in this study (IL-4, IL-6, IL-10, TNF-α, and IFN-γ) were deliberately selected based on their well-established roles in malaria pathogenesis during pregnancy. These molecules are known to modulate the balance between pro- and anti-inflammatory immune responses, influence placental inflammation, and regulate maternal–fetal immune tolerance. This biologically grounded selection ensures comparability with previous immuno-epidemiological studies in malaria-endemic regions.

## 2. Materials and Methods

### 2.1. Study Setting

This study was conducted in the catchment area of the Nanoro Health and Demographic Surveillance System (HDSS), located approximately 85 km west of Ouagadougou, in the central–west region of Burkina Faso. The surveillance area, established and maintained by the Clinical Research Unit of Nanoro (CRUN) since 2019, includes 24 villages and monitors a population of over 63,000 inhabitants [21]. Malaria transmission in the region is highly seasonal, with peaks occurring during the rainy season from June to October, accounting for nearly 50% of all outpatient consultations during this period. Pregnant women remain particularly vulnerable, with malaria prevalence rates ranging from 24% to over 50%, depending on diagnostic methods. Younger women and those not receiving intermittent preventive treatment with sulfadoxine–pyrimethamine (IPT-SP) are at greater risk. The challenge is further exacerbated by asymptomatic infections, which complicate timely detection [22,23,24,25,26]. According to the 2023 national health report [27], IPT-SP coverage in Nanoro remains suboptimal, with only 38.3% of pregnant women completing the recommended three-dose regimen. Ongoing malaria control strategies in the area focus on increasing IPT-SP uptake and the distribution of insecticide-treated nets (ITNs) to reduce the maternal malaria burden.

### 2.2. Study Design and Population

This study was part of the COSMIC trial (ISRCTN37225929), which evaluated malaria prevention strategies in pregnancy [28]. The design, recruitment procedures, and characteristics of the study population have been extensively described in a previous publication [29]. Briefly, the broader study followed a cohort of thirty-three primigravid women from delivery through three months postpartum. However, the present analysis focuses exclusively on data collected at the time of delivery. This includes immunological assessments, malaria screening, and placental evaluations. All participants were recruited from the control group of the COSMIC trial (standard IPTp-SP regimen) and provided written informed consent prior to enrolment. For this analysis, malaria infection at delivery was defined operationally as the presence of *Plasmodium falciparum* parasites detected by microscopy and/or PCR in maternal peripheral blood collected immediately after childbirth, before placental expulsion. Blood samples were obtained within 30 min after delivery, processed on the same day, and stored at −80 °C until laboratory analysis. This approach ensured that parasitological and immunological measurements reflected maternal infection and immune status at the time of delivery.

### 2.3. Ethical Approval

The institutional review board of Centre Muraz/IRSS granted ethical approval for this study (Reference A007-2014/CEI-CM dated 12 February 2014). The approval was amended on 25 July 2014, and subsequently extended on 29 September 2015, for an additional year of follow-up for women (25 July 2014–25 July 2016).

### 2.4. Sample Collection and Laboratory Procedures

Peripheral venous blood (10 mL) was drawn at each time point into heparinized tubes. Samples were processed within four hours, centrifuged at 1800 rpm for 10 min, and stored at −80 °C for subsequent cytokine analysis. Dried blood spots were collected on filter paper for DNA extraction and PCR. Hemoglobin concentration was measured using a Hemocue^®^ Hb 301 device (HemoCue AB; Ängelholm; Sweden). Placental biopsy specimens (2 × 2 × 1 cm) were collected from the maternal-facing surface of the placenta immediately after delivery, fixed in 10% buffered formalin, and embedded in paraffin for histopathology.

Placental samples were successfully collected and analyzed for 21 of the 33 enrolled participants. The difference in sample numbers was due to logistical and technical constraints encountered at delivery, including (i) incomplete sample collection in some cases where placental blood could not be obtained before tissue fixation or expulsion, and (ii) variable sample quality, where certain placental smears or lysates were excluded due to contamination or insufficient material for molecular or immunological assays. These exclusions were made following predefined quality control criteria, ensuring that only samples meeting minimum analytical standards were retained for final analyses.

### 2.5. Malaria Diagnosis

Malaria screening was conducted during antenatal care (ANC) visits at peripheral health facilities (Centres de Santé et de Promotion Sociale, CSPS) in accordance with Burkina Faso’s National Malaria Control Program (NMCP) guidelines. Detection relied on PfHRP2-based rapid diagnostic tests (RDTs) (SD BIOLINE Malaria Ag P.f^®^, Standard Diagnostics Inc., Yongin, Republic of Korea) and microscopy. Testing was performed only after obtaining written informed consent, and RDTs were considered positive when both control and test lines were visible.

Microscopy procedures were performed using Olympus CX23 and CX31RBSF microscopes (Olympus Corporation, Tokyo, Japan), maintained under certified calibration at the Clinical Research Unit of Nanoro. Thick and thin blood smears were stained with 10% Giemsa for 45 min and examined at 100× oil immersion objective (1000× total magnification), following WHO standards. Each thick film was screened for quality, and at least 100 high-power fields (HPF) were examined before a slide was declared negative. If parasites were detected, an additional 100 HPF were examined to confirm *Plasmodium* species and exclude mixed infections.

Slides were examined independently by two blinded expert microscopists, with discrepancies resolved by a third reader. Parasite density was calculated per 200 leukocytes (or 500 when <10 parasites were seen). Microscopy-positive cases were defined as those with ≥1 asexual *P. falciparum* parasite per 200 HPF (≈50 parasites/µL), in line with WHO recommendations.

PCR-positive cases were determined by successful amplification of the *msp2* gene, confirming submicroscopic infections. Infection status at delivery was established using a composite diagnostic approach combining microscopy and nested PCR results from maternal peripheral blood collected immediately after childbirth. Participants were classified as infected if either method tested positive, ensuring inclusion of both microscopic and submicroscopic infections.

### 2.6. PCR-Based Malaria Detection

Genomic DNA was extracted from dried blood spots (DBS) using the QIAamp DNA Mini Kit (QIAGEN, Valencia, CA, USA), following the manufacturer’s instructions. Extracted samples were stored at –20 °C until further processing. To assess *P. falciparum* genetic diversity, we performed a nested PCR targeting the *msp2* gene, using a total reaction volume of 25 µL. For the first round, 5 µL of DNA extract was used, followed by 1 µL of product in the second round with family-specific primers as previously described [30,31]. PCR amplification involved an initial denaturation at 94 °C for 5 min, followed by 36 cycles of denaturation at 94 °C for 1 min, annealing at 58 °C for 2 min, and extension at 72 °C for 2 min, with a final elongation step at 72 °C for 10 min. Amplified fragments were separated by agarose gel electrophoresis, stained with ethidium bromide, and visualized under UV light. Fragment sizes were estimated using Photo CaptMW software (v11.01). Positive and negative controls were included in each PCR run to validate assay performance and exclude contamination. All PCR steps were performed in separate, dedicated work areas using filter tips to minimize cross-contamination risk.

The nested PCR assay targeted the polymorphic region of the *merozoite surface protein-2* (*msp2*) gene, a widely used molecular marker for *P. falciparum* due to its high allelic diversity. This gene was selected for its superior sensitivity and reliability in confirming parasite detection, especially in high-transmission areas where mixed infections are common. The assay was optimized for sensitive detection of *P. falciparum* infection rather than for complete genotyping or clone differentiation and therefore was not intended to assess multiplicity of infection or to distinguish between new and persistent infections.

The nested PCR assay targeting the *msp2* gene was used for parasite detection, not genotyping or allelic diversity analysis. This assay was optimized for sensitive identification of *P. falciparum* DNA and enabled detection of low-density parasitemia that might be missed by microscopy or RDT.

In cases of discordant results (e.g., RDT-positive but microscopy- and PCR-negative), infection classification followed a hierarchical rule favoring molecular confirmation.

- PCR-positive: considered infected
- PCR-negative but microscopy-positive: considered infected
- RDT-positive only: considered non-infected (likely false positive)

The nested PCR assay targeting the *msp2* gene achieved a limit of detection (LOD) of approximately 5 parasites/μL, allowing the identification of submicroscopic infections undetectable by microscopy. Each DNA sample was analyzed in duplicate in independent PCR runs, and discordant results were re-tested to confirm reproducibility. Positive and negative controls were included in every batch to ensure assay consistency across runs.

### 2.7. Immunological Biomarker Quantification

#### 2.7.1. Cytokine Quantification

Cytokine concentrations in plasma were analyzed to assess the inflammatory and regulatory immune environment at delivery [32]. Blood samples were centrifuged at 1800 rpm for 10 min, and plasma was stored at −80 °C until analysis. Commercial ELISA kits validated for human cytokine detection were used (eBioscience BMS225/2 for IL-4, BioSource Europe KAC1261 for IL-6, Invitrogen KHC0101 for IL-10, Invitrogen KHC3011 for TNF-α, and Invitrogen KAC1231 for IFN-γ), following manufacturer protocols. Standard curves were generated using recombinant cytokines supplied in each kit, and plasma samples were analyzed in duplicate. Detection was achieved using biotin-conjugated detection antibodies, streptavidin–horseradish peroxidase (HRP), and tetramethylbenzidine (TMB) substrate. Optical density was read at 450 nm (reference 570 nm) using a Multiskan™ GO reader (Thermo Fisher Scientific, Waltham, Country).

Each cytokine assay followed the manufacturer’s specifications for analytical sensitivity and range. For IFN-γ (Invitrogen KAC1231), concentrations were reported in IU/mL, as defined by the manufacturer’s WHO-standardized calibration curve [32]. The numeric IFN-γ values therefore represent IU-equivalent concentrations derived from the standard curve rather than pg/mL measurements. Values falling below the assay’s lower limit of quantification (0.03 IU/mL) appeared as low-level background-corrected optical-density signals after blank subtraction. These sub-quantification values were retained in the dataset to preserve distributional structure for multivariate analyses; however, they should not be interpreted as precise cytokine concentrations. Quantifiable cytokine concentrations fell within the validated dynamic ranges specified by each manufacturer. For cytokines with values below the lower limit of quantification, the retained measurements represent background-corrected optical-density residuals rather than precise concentrations. These sub-quantification values were included to preserve the full variance structure required for multivariate analyses and are interpreted accordingly. The cytokine panel (IL-4, IL-6, IL-10, TNF-α, IFN-γ) was selected for its established role in *P. falciparum* infection, maternal immune modulation, and malaria-related inflammation during pregnancy.

#### 2.7.2. Antibody Quantification

Plasma IgG antibody responses to three *Plasmodium falciparum* antigens, *Pf*CSP (circumsporozoite protein), *Pf*AMA-1 (apical membrane antigen 1), and EBA-175 (erythrocyte binding antigen), were measured by enzyme-linked immunosorbent assay (ELISA). Antibody responses were quantified as optical density (OD) values measured at 450 nm (OD_450_), rather than as endpoint titers. These antigens were selected to represent key stages of the parasite life cycle: sporozoite (*Pf*CSP), merozoite invasion (*Pf*AMA-1), and erythrocyte binding (EBA-175).

High-binding 96-well ELISA plates were coated overnight at 4 °C with 1 µg/mL of each recombinant antigen in carbonate–bicarbonate buffer (pH 9.6). Plates were then blocked with 5% non-fat dry milk in PBS-Tween (0.05%) for 1 h at room temperature. Plasma samples were diluted at 1:100 in blocking buffer and added in duplicate. Following incubation and washing, bound antibodies were detected using horseradish peroxidase-conjugated goat anti-human IgG (Life Technologies, Carlsbad, CA, USA, A24470), and color development was achieved using TMB substrate. Reactions were stopped with 1 M H_2_SO_4_, and absorbance was read at 450 nm. A positive control (pooled plasma from hyperimmune multigravid women in Nanoro) and a negative control (plasma from malaria-naïve Caucasian donors) were included on each plate.

All OD_450_ readings were background-corrected by subtracting the mean optical density of blank wells (buffer without antigen). To account for inter-plate variability, background-corrected OD values were normalized to the pooled positive control included on each plate and expressed as relative OD ratios (sample OD/positive control OD). These normalized continuous OD ratios were used for all downstream quantitative analyses, including principal component analysis (PCA) and hierarchical clustering. Seropositivity for each antigen was defined as a normalized OD ratio exceeding the mean + 2 standard deviations of the negative control sera derived from malaria-naïve donors. While seropositivity thresholds were used for descriptive purposes, continuous normalized OD ratios were retained for multivariate analyses to preserve quantitative immune variation.

### 2.8. Histological Assessment of Placental Malaria

Placental histology was performed for descriptive and comparative purposes only and did not influence the definition of infection at delivery. Infection classification was based solely on maternal peripheral blood results from microscopy and PCR. At delivery, placental biopsy samples approximately 2 × 2 × 1 cm in size were collected from the maternal surface and immediately fixed in 10% neutral buffered formalin to preserve tissue integrity. Samples were then processed and embedded in paraffin wax for histological evaluation. Thin sections were stained with hematoxylin and eosin and examined by experienced microscopists blinded to clinical outcomes. Based on standard morphological criteria described by Bulmer et al., placental malaria was classified into four categories: acute infection (presence of parasites without malaria pigment), chronic infection (parasites and pigment present), past infection (no parasites or pigment), and no infection (absence of both parasites and pigment) [33].

### 2.9. Data Analysis

#### 2.9.1. Statistical Analyses

All statistical analyses were conducted using R version 4.3.1 (R Foundation for Statistical Computing) and GraphPad Prism version 9.5 (GraphPad Software, San Diego, CA, USA). Descriptive statistics were used to summarize demographic characteristics, clinical variables, and immunological biomarkers. Continuous variables were reported as means with standard deviations (SD) or medians with interquartile ranges (IQR), depending on the underlying data distribution. Categorical variables were summarized as frequencies and percentages. Given the limited sample size (*n* = 33), all analyses were treated as exploratory and hypothesis-generating. No multivariable modeling was applied beyond unsupervised clustering, as the aim was to describe immune heterogeneity rather than infer causality. Associations between immune profiles and malaria infection were further examined using Fisher’s exact test to assess infection distribution across clusters. Because this was a cross-sectional study, cytokine–infection relationships were interpreted as concurrent associations at delivery, without implying predictive or causal inference.

All cytokine concentration data (IL-4, IL-6, IL-10, TNF-α, IFN-γ) were used as originally measured. For cytokines quantified in pg/mL (IL-4, IL-6, IL-10, TNF-α), values below the assay-specific lower limit of detection reflected background-corrected optical-density residuals after blank subtraction. For IFN-γ, sub-quantification values (<0.03 IU/mL) similarly represented residual OD signals rather than measurable concentrations. Accordingly, values below quantification are treated analytically as low-level residual signals rather than true cytokine concentrations.

#### 2.9.2. Positivity Definition for Cytokine and Antibody Responses

Cytokine and antibody positivity thresholds were defined as values exceeding the mean plus two standard deviations (mean + 2 SD) of the uninfected control group. This statistical criterion is widely used in malaria immuno-epidemiological research to differentiate baseline immune variation from biologically elevated responses associated with infection. Comparable approaches have been applied in both controlled human malaria infection (CHMI) studies and endemic field settings, where the 2 SD rule is recognized for its balance between sensitivity and specificity [34,35,36,37].

The uninfected reference group used to establish this threshold consisted of malaria-negative women from the same population, ensuring comparable immunological background and exposure history. This criterion was selected to minimize false positives and maintain methodological consistency with previous sero-surveillance and cytokine profiling studies in *Plasmodium falciparum*-endemic areas.

#### 2.9.3. Principal Component Analysis (PCA)

**Figure 1:**
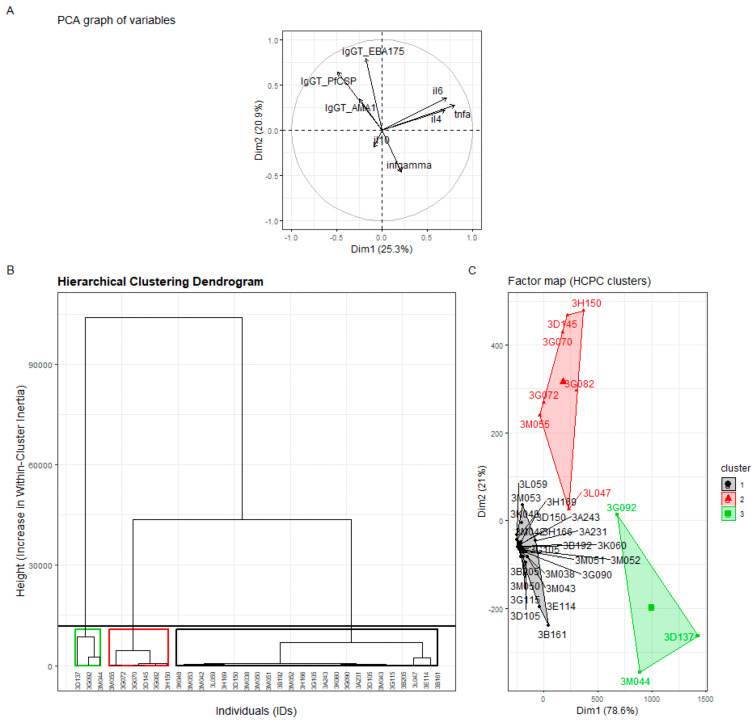
Principal component and hierarchical clustering analyses revealing three distinct cytokine-driven immune phenotypes among primigravid women at delivery. (**A**) PCA biplot showing individual distribution along the first two principal components (Dim 1 = 38.6%, Dim 2 = 32.4%). (**B**) Dendrogram generated from hierarchical clustering of PCA scores using Ward’s linkage and Euclidean distance; the y-axis represents Euclidean distance indicating relative immunological dissimilarity between participants. (**C**) Heatmap of standardized (z-score normalized) cytokine and antibody concentrations ordered by HCA cluster assignment. Clusters (C1–C3) are color-coded consistently across panels, highlighting distinct cytokine gradients with Cluster 3 showing a markedly pro-inflammatory profile.

To identify latent immune profiles, principal component analysis (PCA) was performed on the complete panel of immunological biomarkers, including cytokine levels (IL-4, IL-6, IL-10, TNF-α, IFN-γ) and antibody responses (*Pf*AMA-1, *Pf*CSP, EBA-175). Biomarker values were not log-transformed. Prior to PCA, all biomarker variables were mean-centered and scaled to unit variance (z-score normalization). Hierarchical clustering (Ward’s linkage; Euclidean distance) was then applied to the PCA score matrix, retaining all principal components to preserve the full variance structure. The number of clusters was selected using the inertia (elbow) criterion and confirmed by inspection of the dendrogram (k = 3). For secondary clinical variables, participants with incomplete data were excluded from PCA/HCA. Cluster stability was evaluated by bootstrap resampling (1000 iterations) using pvclust, with AU values ranging from 92 to 98 cytokine and antibody variables were used in their original units (pg/mL or IU/mL for IFN-γ), then centered and scaled (z-score normalization) prior to PCA. Hierarchical clustering was then applied to the PCA scores using Ward’s linkage method and Euclidean distance to stratify participants into immunologically distinct clusters. Hierarchical clustering was applied to the PCA score space, and the resulting dendrogram (Figure 1B) displays Euclidean distances between participants’ PCA coordinates (branch length on the y-axis). Cluster membership used throughout the manuscript was defined from this HCA-on-PCA workflow, and the same PCA-derived cluster assignments were used for all downstream comparisons. These clusters were interpreted as immune profiles and used for subsequent analyses.

Hierarchical clustering was performed on the full set of PCA scores, retaining all principal components to preserve the complete variance structure of the biomarker dataset. Using all PCs avoids arbitrary dimensionality reduction and ensures that clustering reflects the integrated multivariate relationships among cytokines and antibody responses. This sequential PCA–HCA approach was implemented to reduce data dimensionality, minimize noise, and improve the biological interpretability of resulting immune profiles. Cluster classification was therefore based on HCA of PCA scores, rather than raw cytokine concentrations, to ensure that patterns reflected integrated multivariate immune relationships. The dendrogram’s y-axis represents Euclidean distance calculated from the PCA-transformed data, indicating relative immunological dissimilarity among participants. All subsequent comparative analyses, including the assessment of malaria infection distribution across clusters, were performed using these same PCA-derived cluster assignments. Cluster-specific infection frequencies were calculated using these PCA-HCA classifications to evaluate associations between immune phenotypes and malaria infection at delivery.

To explore immune heterogeneity at the individual level, we created a heatmap based on scaled concentrations of eight selected immune biomarkers: *Pf*CSP, *Pf*AMA-1, EBA-175, IL-4, IL-10, TNF-α, IL-6, and IFN-γ. Each row represented a participant, and each column corresponded to a specific biomarker. Color gradients reflected the relative expression levels, making it easier to visually detect patterns of immune responses across different clusters. Before visualization, all biomarker values were standardized using z-score transformation. This z-score scaling (mean-centering and division by the standard deviation) allowed comparison of biomarkers with different concentration ranges and reduced the influence of extreme values. Samples on the heatmap were ordered according to their HCA-derived cluster membership, ensuring that cluster-specific cytokine expression patterns could be visually distinguished. Cluster labels (C1–C3) were color-coded consistently between the PCA and heatmap representations to enhance interpretability.

Associations between immune profiles and clinical characteristics including malaria infection at delivery, anemia, number of IPTp-SP doses, season of delivery, and placental malaria were evaluated using chi-square or Fisher’s exact tests for categorical variables, and ANOVA or Kruskal–Wallis tests for continuous variables, as appropriate based on distribution. Special emphasis was placed on comparing malaria infection rates across clusters to explore potential links between immune profile and susceptibility. Given the exploratory nature of the study, no corrections for multiple comparisons were applied. Statistical significance was set at *p* < 0.05.

#### 2.9.4. Cluster Stability via Bootstrapping

To test how reliable the immune profiles were after unsupervised clustering, we carried out a cluster stability assessment using non-parametric bootstrapping. We applied hierarchical clustering to scaled data from eight key immunological biomarkers: three antibody titers (*Pf*AMA-1, *Pf*CSP, and EBA-175) and five cytokines (IL-4, IL-6, IL-10, TNF-α, and IFN-γ). The clustering method used Ward’s linkage and Euclidean distance within a PCA-transformed feature space.

We measured cluster stability using the pvclust algorithm, which runs 1000 bootstrap resampling iterations and reports two key metrics:

- The approximately unbiased (AU) *p*-value, based on multiscale bootstrap resampling
- The Bootstrap Probability (BP), based on standard resampling

Clusters with AU *p*-values of 95% or higher were deemed statistically stable. This validation step ensured the internal consistency of the immune profiles identified by the clustering algorithm.

Although the sample size (*n* = 33) was limited, it was deemed appropriate for an exploratory multivariate approach focused on identifying cytokine-driven immunological patterns. The variable-to-sample ratio met standard criteria for unsupervised analyses. To evaluate the robustness of the clustering results and minimize overfitting, hierarchical clustering stability was assessed through 1000 bootstrap iterations using the pvclust RStudio (version 2024.12.0, Posit Software, PBC, Boston, MA, USA). The resulting approximately unbiased (AU) *p*-values (≥92%) confirmed high internal stability of the three-cluster solution.

## 3. Results

### 3.1. Demographic Characteristics

A total of 33 primigravid women were enrolled, with a mean age of 19 years (SD = 1.6; range 18–20.5 years). Anemia (Hb ≤ 11 g/dL) was observed in 60.6% of participants, and nearly half (48.5%) were infected with *Plasmodium falciparum* at delivery. Slightly more than half (51.5%) had received ≤2 IPTp-SP doses during their pregnancy. The mean parasite density among microscopy-positive cases was 540.9 parasites/μL (range: 0–5584). This value represents the average parasite burden among microscopy-detectable infections, not the assay’s detection limit, which was approximately 50 parasites/μL for microscopy and 5 parasites/μL for nested PCR. Among the 21 participants with placental samples, only 4.8% had active placental malaria, 19.1% had past infections, and 76.2% had no evidence of infection. Dystocia was observed in 18.2% of deliveries. Most deliveries (60.6%) occurred during the high-transmission season (Table 1).

### 3.2. Descriptive Statistics of Immunological Biomarkers Immediately After Delivery

Plasma antibody titers and cytokine concentrations varied widely across individuals (Table 2). All cytokine concentrations reported in this study were within the validated detection ranges for their respective assays. Mean antibody levels were PfAMA-1 (0.625 ± 0.380), EBA-175 (2.160 ± 1.164), and PfCSP (1.328 ± 0.997).

Cytokine levels showed a predominance of pro-inflammatory mediators: IL-6 (186.87 ± 328.06 pg/mL) and TNF-α (189.69 ± 270.02 pg/mL), compared with lower IFN-γ (0.035 ± 0.029 pg/mL).

### 3.3. Immunological Profiles of the Study Population Independently to Their Malaria Status

Principal component analysis (PCA) applied to the eight immunological biomarkers revealed three distinct immune clusters (Profiles 1–3), together explaining 71% of total variance. The first two principal components were primarily driven by IL-6 and TNF-α, which clearly separated participants with pro-inflammatory and low-inflammatory individuals (Figure 1A–C).

Cluster 1 represented a low-inflammatory profile, characterized by low IL-6 and TNF-α concentrations. Cluster 2 corresponded to an intermediate-inflammatory or TNF-α-dominant profile, with elevated TNF-α and relatively low IL-6. Cluster 3 defined a high-inflammatory profile, marked by concurrent elevation in IL-6 and TNF-α. These cluster definitions are provided here and in Figure 1 legend to facilitate immediate interpretation of the data.

Cluster assignment was statistically robust. Bootstrap resampling (1000 iterations) using the pvclust method yielded approximately unbiased (AU) *p*-values of 98%, 92%, and 96% for Clusters 1, 2, and 3, respectively, confirming the internal stability and biological consistency of the three-cluster solution.

**Figure 2:**
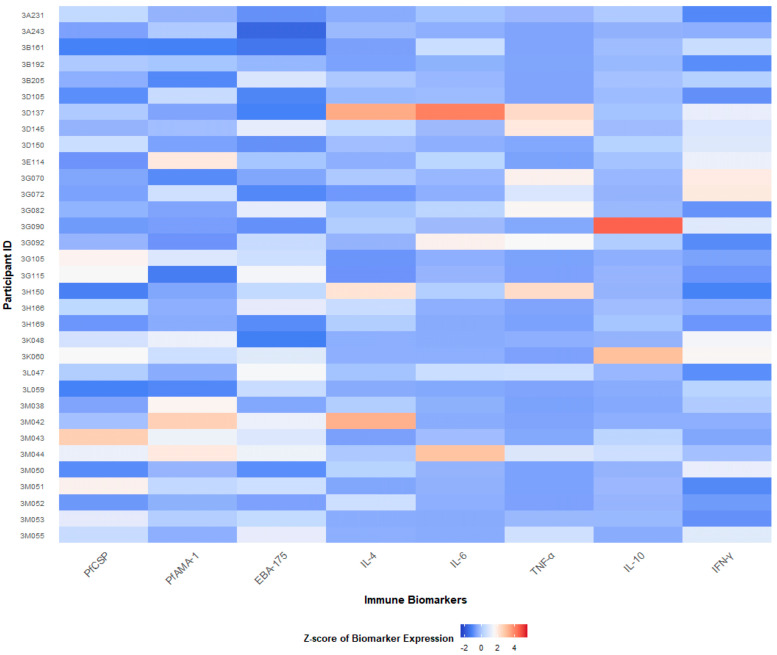
Heatmap of immune biomarker expression across study participants. Columns represent biomarkers (PfCSP, PfAMA-1, EBA-175, IL-4, IL-10, IL-6, TNF-α, IFN-γ), while rows represent participants grouped by hierarchical cluster assignment. The color scale reflects standardized (z-score) expression values, with red indicating higher and blue indicating lower relative concentrations.

To visualize these findings at the participant level, a heatmap of cytokine and antibody expression was generated (Figure 2). Distinct cytokine-driven signatures emerged: Cluster 3 participants displayed markedly elevated IL-6 and TNF-α levels, consistent with a hyper-inflammatory phenotype, while Cluster 1 individuals exhibited low concentrations of these cytokines. In contrast, antibody responses to *P. falciparum* antigens (PfCSP, PfAMA-1, EBA-175) did not clearly differentiate among clusters, suggesting that cytokine-based profiles provide a more discriminative immunological signature in this population.

The first profile primarily included individuals with low levels of IL-6 and TNF-α, indicative of a subdued pro-inflammatory response (Table 3).

The second profile consisted of individuals with elevated levels of TNF-α, suggesting a heightened pro-inflammatory state (Table 4).

The third profile mainly comprised individuals with elevated levels of both IL-6 and TNF-α, reflecting a strong pro-inflammatory response (Table 5).

### 3.4. Association of Immunological Profiles with Malaria Status of Primigravid Women

Following the identification of three distinct immune clusters (C1–C3) derived from the PCA and hierarchical clustering analyses, as described in Table 6. We next evaluated the distribution of *Plasmodium falciparum* infection across these clusters. This analysis aimed to determine whether the cytokine-driven immune phenotypes were associated with infection status at delivery. No additional PCA was performed at this stage; all analyses were based on the cluster classification established from the initial PCA.

The distribution of *P. falciparum* infection across clusters is presented in Table 6. Cluster 1 exhibited a low-inflammatory cytokine profile characterized by low IL-6 and TNF-α concentrations, with 47.8% of participants infected at delivery. Cluster 2 showed an intermediate-inflammatory pattern, defined by elevated TNF-α but low IL-6, with a lower infection proportion (28.6%). Cluster 3 presented a highly pro-inflammatory phenotype, marked by concurrent elevation of IL-6 and TNF-α, and all individuals (100%) in this group were infected at delivery.

These findings demonstrate a graded relationship between inflammatory intensity and infection prevalence. Specifically, infection frequency increased progressively from Cluster 1 (low-inflammatory) through Cluster 3 (highly pro-inflammatory). Cluster 2, although characterized by elevated TNF-α levels, showed a lower infection proportion, highlighting that not all inflammatory patterns correspond uniformly to infection. These results should therefore be interpreted as descriptive of immune activation at delivery rather than predictive of postpartum malaria. Because samples were collected only at the time of delivery, the immune phenotypes identified here represent concurrent infection-associated inflammatory responses. No postpartum follow-up data were used to determine whether these cytokine profiles persist or normalize after childbirth.

Although all participants in Cluster 3 were malaria-positive at delivery, this observation is based on a very small subgroup (*n* = 3) and should therefore be interpreted with caution. The apparent association between cytokine-driven clusters and malaria infection status is exploratory and hypothesis-generating, requiring confirmation in larger, independent studies. The corresponding odds ratio (OR = 9.0; 95% CI: 0.66–∞) is reported to illustrate the wide uncertainty surrounding this estimate.

Specifically, all three participants in Cluster 3, the highly pro-inflammatory group characterized by markedly elevated IL-6 and TNF-α, were infected at delivery, while Cluster 1 predominantly consisted of non-infected individuals showing low cytokine levels. Cluster 2, with intermediate TNF-α expression, exhibited a mixed infection profile.

**Figure 3:**
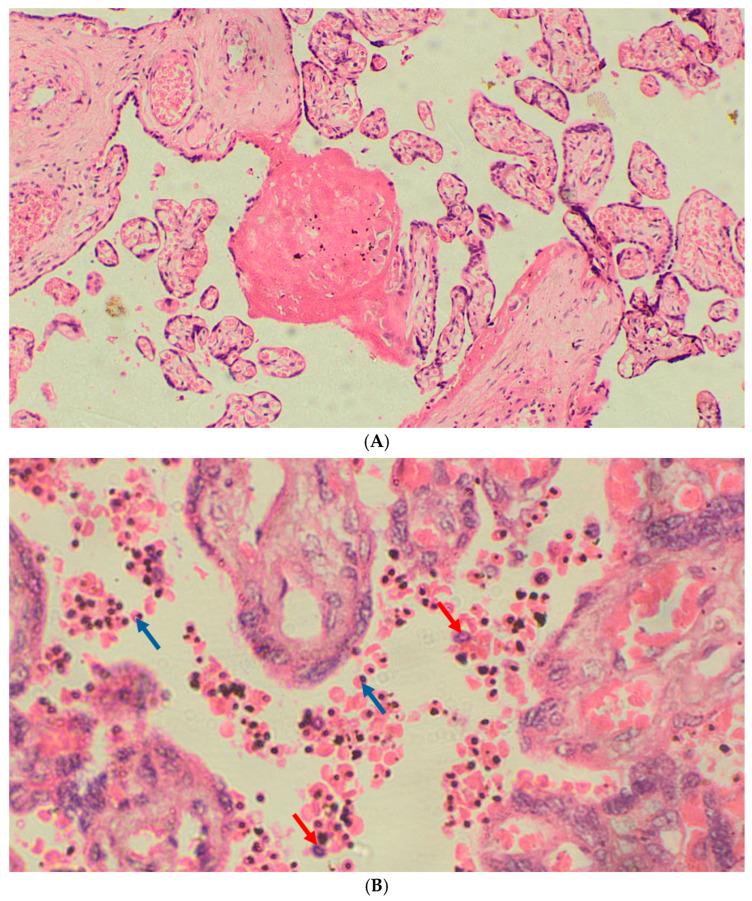
Representative histopathological features of placentas at delivery. Blue arrows indicate presence of infected erythrocytes. Red arrows indicate leucocytes with parasite pigment. (**A**) Normal placenta showing preserved villous architecture and absence of parasitized erythrocytes or pigment. (**B**) Placenta from a woman with active *P. falciparum* infection at delivery, characterized by parasitized erythrocytes and hemozoin pigment deposition in intervillous spaces (H&E stain, ×1000).

Representative histological examinations of placental tissue collected at delivery revealed clear morphological distinctions between non-infected and infected cases. Placentas from malaria-negative women displayed normal villous architecture and absence of parasites or pigment (Figure 3A), whereas infected placentas exhibited *Plasmodium falciparum*-infected erythrocytes, hemozoin pigment deposition, and mononuclear cell infiltration consistent with active placental malaria (Figure 3B). These observations provide qualitative context to the immunological profiles identified in this study but were not directly integrated into the clustering analysis.

These findings suggest that cytokine-driven immune activation at delivery mirrors concurrent malaria infection rather than reflecting predisposition to future infection. The association between the most inflammatory phenotype and active *P. falciparum* infection underscores the biological relevance of IL-6 and TNF-α as markers of infection-related inflammation at the time of childbirth.

Given the cross-sectional nature of the analysis, these associations should be interpreted as descriptive of infection-related inflammation at the time of delivery, not as predictors of postpartum malaria.

## 4. Discussion

This exploratory immunoprofiling study identified three biologically distinct cytokine-driven immune phenotypes among primigravid women at delivery. Using principal component and clustering analyses, we observed that pro-inflammatory profiles (elevated IL-6 and TNF-α) were consistently associated with active *P. falciparum* infection. Malaria infection status was interpreted in relation to the immune phenotypes defined by the initial PCA-based clustering. These findings are hypothesis-generating and indicate that elevated IL-6 and TNF-α levels in the high-inflammatory phenotype (Cluster 3) are associated with *P. falciparum* infection at delivery, reflecting concurrent immune activation rather than predictive susceptibility. As this study analyzed samples collected exclusively at delivery, the cytokine profiles represent infection-associated immune responses at childbirth.

Given the strong pro-inflammatory nature of IL-6 and TNF-α, the clustering patterns observed most likely reflect infection-driven immune activation coinciding with malaria infection, rather than intrinsic predisposition. This interpretation aligns with the cross-sectional design of the study, which captures immune responses at a single time point and cannot establish causality or temporal susceptibility. Consequently, these findings should be viewed as descriptive of infection-associated inflammation, not as indicative of pre-existing immune bias. Cytokine profiles should therefore be interpreted as correlates of infection at delivery, not as indicators of postpartum risk. The observed elevations in IL-6 and TNF-α likely represent acute inflammatory activation coinciding with malaria infection at delivery. Given the cross-sectional design, it is not possible to determine whether these cytokine-responses reflect causal pathways of susceptibility or are consequences of active infection. Additionally, labor-associated inflammation and concurrent bacterial or viral infections may also contribute to the cytokine elevations observed. These alternative explanations have been considered, and the findings are therefore interpreted as reflecting concurrent immune activation rather than predictive immune predisposition. Because IFN-γ was quantified in IU/mL and sub-quantification values represented residual OD signals rather than measurable concentrations, interpretations involving IFN-γ should be considered exploratory.

In particular, the observed clustering association between the high-inflammatory phenotype (Cluster 3) and malaria infection should be regarded as descriptive rather than inferential, given the limited number of individuals (*n* = 3) within that subgroup. The small sample size restricts statistical power and generalizability, underscoring the exploratory nature of this finding. Future longitudinal studies are warranted to determine whether these cytokine patterns persist beyond delivery or resolve following infection clearance.

Despite these limitations, the identified clusters exhibited strong statistical robustness, with bootstrap support values of 98%, 92%, and 96%, respectively, suggesting that the observed immunological patterns reflect true biological variation rather than random noise.

Notably, all women in the most inflammatory group (Cluster 3) were infected at delivery, highlighting a potential link between heightened cytokine activation and concurrent parasitemia rather than predictive risk beyond this time point. This heterogeneity was further illustrated in a participant-level heatmap, where clear differences in cytokine expression patterns were observed across clusters. Collectively, these results underscore the immunological diversity among primigravidae at delivery and suggest that integrated cytokine signatures may capture infection-related immune activation more accurately than single biomarkers.

A graded pattern was observed in infection prevalence across clusters, with 47.8% infection in Cluster 1 (low-inflammatory), 28.6% in Cluster 2 (TNF-α-dominant), and 100% in Cluster 3 (high-inflammatory). This confirms that malaria infection at delivery is most strongly associated with the hyper-inflammatory phenotype rather than intermediate cytokine activation, reinforcing the biological consistency of our clustering results.

The pro-inflammatory clusters identified align with earlier studies emphasizing the role of cytokines in malaria pathogenesis during pregnancy. Moormann et al. [38] reported elevated TNF-α and IFN-γ in placental malaria cases associated with intrauterine growth retardation (IUGR) and adverse pregnancy outcomes. Our findings extend this evidence by showing that women in Cluster 3, characterized by concurrent elevations in IL-6 and TNF-α, were exclusively infected at delivery. This suggests that combined cytokine elevation may serve as a more accurate immunological signature of infection-associated inflammation during parturition.

Interestingly, antibody responses to *P. falciparum* antigens (*Pf*CSP, *Pf*AMA-1, EBA-175) did not differentiate between clusters, indicating that humoral immunity alone may not sufficiently capture immunological vulnerability. This observation is consistent with prior reports showing that high antibody titers do not necessarily confer clinical protection, particularly in primigravidae with limited prior exposure [14]. Thus, cellular and cytokine-mediated mechanisms may provide a more informative framework for stratifying infection status and inflammatory burden in malaria-endemic populations.

IL-10, an anti-inflammatory cytokine, was variably expressed across clusters. Cluster 3 individuals exhibited elevated IL-10 alongside TNF-α, potentially reflecting a compensatory regulatory response to hyperinflammation. Othoro et al. [39] proposed that a low IL-10/TNF-α ratio is linked to severe malaria-related anemia. Although our dataset did not explicitly explore this ratio, future investigations could evaluate whether shifts in these cytokine balances modulate maternal immune resilience and recovery after infection.

From a methodological perspective, the combined use of principal component analysis (PCA) and hierarchical clustering allowed the identification of latent immune phenotypes that conventional univariate tests may overlook. This systems-level approach aligns with prior immunoprofiling frameworks applied in vaccine and maternal health research [14].

We acknowledge that the present analysis was performed on a targeted cytokine panel and therefore represents a partial view of the immune landscape. While this approach enhances interpretability and focuses on biologically validated markers, untargeted discovery-based methods such as proteomics, transcriptomics, or multiplex inflammatory panels could provide deeper insights into uncharacterized immune pathways. Integrating such high-dimensional approaches in future studies would allow a more comprehensive assessment of immune mechanisms underlying malaria susceptibility in pregnancy. In addition, although the PCR assay amplified the polymorphic region of msp2, it was optimized for infection detection rather than full genotyping. Consequently, the study was not designed to differentiate between new and persistent infections. Future longitudinal studies incorporating detailed msp2 genotyping could help distinguish recrudescent from newly acquired infections and provide a deeper understanding of immune dynamics at delivery, for instance, clarifying whether sustained IL-10 expression reflects lingering parasitaemia carried from pregnancy into the postpartum period.

Though the relatively small sample size (*n* = 33) limits statistical power and generalizability, the variable-to-sample ratio was acceptable for exploratory multivariate analysis. To address potential overfitting, bootstrap resampling (1000 iterations) was performed using the pvclust R package, yielding high approximately unbiased (AU) *p*-values (≥92%) and confirming cluster stability.

The present findings also contribute to the expanding global initiative toward data-driven immunoprofiling and infection risk prediction. Similar computational and translational approaches have been successfully implemented in sepsis and vaccine research contexts [15,17], supporting the broader applicability of integrated biomarker modeling in infectious disease surveillance. By aligning with these international data harmonization efforts [18], this study provides an exploratory step toward standardized immune-response profiling in maternal malaria.

Given the exploratory and cross-sectional design of this study, the findings should be interpreted with appropriate caution. We acknowledge that the small cohort size may limit generalizability and that causality cannot be inferred. Nevertheless, the internal validation procedure supports the robustness of the observed immune clusters and their biological plausibility. The cytokine-driven phenotypes identified here likely reflect infection-associated immune activation at delivery, rather than predisposition to postpartum malaria. Future longitudinal follow-up studies are needed to determine whether delivery cytokine patterns persist postpartum and whether they are associated with subsequent malaria episodes. Accordingly, this work should be regarded as hypothesis-generating and provides a conceptual and methodological foundation for future longitudinal studies designed to evaluate whether cytokine profiles at delivery can serve as reliable predictors of postpartum infection risk or guide targeted preventive interventions.

## 5. Conclusions

This study reveals substantial immunological heterogeneity among primigravidae at delivery, with distinct cytokine profiles that correlate with malaria infection status. These findings highlight immunological heterogeneity at delivery and its association with malaria infection status. Larger longitudinal studies are warranted to evaluate the postpartum relevance of these immune signatures.

## Figures and Tables

**Table 1:** Summary of baseline characteristics among primigravid participants at delivery (*n* = 33).

Characteristic	Value
Age (years), Mean ± SD	19 ± 1.6
Anemia at delivery (Hb ≤ 11 g/dL), *n* (%)	20 (60.6)
IPTp-SP doses, *n* (%)	
≤2 doses	17 (51.5)
>2 doses	16 (48.5)
Peripheral parasite density at delivery, Mean (min–max)	540.9 parasites/µL (0–5584)
Malaria infection at delivery, *n* (%)	16 (48.5)
Placental malaria status (*N* = 21), *n* (%)	
– Active	1 (4.8)
– Past only	4 (19.1)
– No infection	16 (76.2)
Delivery conditions, *n* (%)	
Dystocia	6 (18.2)
Period of delivery, *n* (%)	
High-transmission season	20 (60.6)
Low-transmission season	13 (39.4)

Abbreviations: IPTp-SP, intermittent preventive treatment in pregnancy with sulfadoxine–pyrimethamine; SD, standard deviation. Infection status was defined using composite microscopy/PCR results from maternal peripheral blood collected at delivery.

**Table 2:** Immunological biomarker concentrations among primigravid participants at delivery (*n* = 33).

Biomarker	Mean *	Std. Dev	Q1	Median	Q3
IgGT_*Pf*AMA1	0.625	0.380	0.381	0.477	0.845
IgGT_EBA175	2.160	1.164	1.043	2.567	3.292
IgGT_*Pf*CSP	1.328	0.997	0.540	0.960	1.864
IL-10	15.530	22.507	7.014	9.031	13.128
IL-4	30.999	17.496	21.827	26.277	31.600
IL-6	186.873	328.056	31.919	57.103	153.484
IFN-γ	0.035	0.029	0.009	0.023	0.063
TNF-α	189.689	270.024	10.438	21.656	364.931

Abbreviations: IgGT, total IgG titer; IL, interleukin; IFN-γ, interferon gamma; TNF-α, tumor necrosis factor alpha; Q1 and Q3, first and third quartiles. * Note: All cytokine concentrations were within the validated assay detection ranges (IL-6: 2–2000 pg/mL; TNF-α: 5–1000 pg/mL; IL-10: 3–1000 pg/mL). IFN-γ concentrations are reported in IU/mL according to the manufacturer’s WHO-standardized calibration curve; values below 0.03 IU/mL represent background-corrected OD residuals and are not quantifiable concentrations [32].

**Table 3:** Immunological biomarkers in the cluster 1.

	Mean **	Std. Dev	Q1	Median	Q3
IgGT_AMA1	0.665	0.399	0.372	0.595	0.954
IgGT_EBA175	1.967	1.133	0.996	1.775	3.073
IgGT_*Pf*CSP	1.411	1.114	0.473	1.174	2.318
IL-10	17.463	26.550	6.812	9.155	13.966
IL-4	28.171	14.549	20.440	23.454	31.600
IL-6 *	75.910	85.140	27.608	47.585	95.485
IFN-γ	0.032	0.026	0.011	0.022	0.062
TNF-α *	25.439	30.029	7.838	15.044	28.751

*: Higher values were observed for IL-6 and TNF-α. ** Note: All cytokine concentrations were within the validated assay detection ranges (IL-6: 2–2000 pg/mL; TNF-α: 5–1000 pg/mL; IL-10: 3–1000 pg/mL). IFN-γ concentrations are reported in IU/mL according to the manufacturer’s WHO-standardized calibration curve; values below 0.03 IU/mL represent background-corrected OD residuals and are not quantifiable concentrations [32].

**Table 4:** Immunological biomarkers in the cluster 2.

	Mean **	Std. Dev	Q1	Median	Q3
IgGT_AMA1	0.472	0.194	0.382	0.434	0.550
IgGT_EBA175	2.712	1.130	1.458	3.399	3.415
IgGT_*Pf*CSP	0.935	0.530	0.673	0.952	1.371
IL-10	7.674	1.812	6.998	8.410	8.674
IL-4	33.127	15.944	22.497	29.439	37.097
IL-6	152.564	129.522	31.178	95.791	263.601
IFN-γ	0.045	0.038	0.007	0.058	0.087
TNF-α *	551.411	177.349	364.931	605.935	698.965

*: Higher value was observed for TNF-α. ** Note: All cytokine concentrations were within the validated assay detection ranges (IL-6: 2–2000 pg/mL; TNF-α: 5–1000 pg/mL; IL-10: 3–1000 pg/mL). IFN-γ concentrations are reported in IU/mL according to the manufacturer’s WHO-standardized calibration curve; values below 0.03 IU/mL represent background-corrected OD residuals and are not quantifiable concentrations [32].

**Table 5:** Immunological biomarkers in Cluster 3.

	Mean **	Std. Dev	Q1	Median	Q3
IgGT_AMA1	0.669	0.582	0.289	0.380	1.339
IgGT_EBA175	2.348	1.479	0.741	2.646	3.656
IgGT_*Pf*CSP	1.606	0.833	0.960	1.312	2.546
IL-10	19.039	7.862	13.128	16.029	27.962
IL-4	47.721	35.773	24.123	30.159	88.881
IL-6 *	1117.641	382.957	736.716	1113.609	1502.598
IFN-γ	0.035	0.031	0.005	0.031	0.068
TNF-α *	604.922	188.436	421.158	595.901	797.706

*: Higher values were observed for IL-6 and TNF-α. ** Note: All cytokine concentrations were within the validated assay detection ranges (IL-6: 2–2000 pg/mL; TNF-α: 5–1000 pg/mL; IL-10: 3–1000 pg/mL). IFN-γ concentrations are reported in IU/mL according to the manufacturer’s WHO-standardized calibration curve; values below 0.03 IU/mL represent background-corrected OD residuals and are not quantifiable concentrations [32].

**Table 6:** Distribution of *Plasmodium falciparum* infection status across immunological clusters at delivery.

Cluster	Malaria Status *		Total
	Infected	Non-Infected	
Profile 1—(*n*, %)	11 (47.83)	12 (52.17)	23 (69.70)
Profile 2—(*n*, %)	2 (28.57)	5 (71.43)	7 (21.21)
Profile 3—(*n*, %)	3 (100.00)	0 (0.00)	3 (9.09)
Total—(*n*, %)	16 (48.48)	17 (51.52)	33 (100)

* Infection status was defined using composite microscopy/PCR results from maternal peripheral blood collected at delivery. Statistical association was evaluated using Fisher’s exact test (*p* = 0.04).

## Data Availability

All data generated and analyzed during this study are included in this published article. Raw data supporting the findings of this study are available from the corresponding author on request.

## Abbreviations

The following abbreviations are used in this manuscript:

IL	Interleukin
TNF-α	Tumor Necrosis Factor-alpha
IFN-γ	Interferon-gamma
IPTp-SP	Intermittent Preventive Treatment in Pregnancy with Sulfadoxine–Pyrimethamine
*Pf*CSP	*Plasmodium falciparum* Circumsporozoite Protein
*Pf*AMA-1	*Plasmodium falciparum* Apical Membrane Antigen 1
EBA-175	Erythrocyte Binding Antigen-175
DBS	Dried Blood Spot
ELISA	Enzyme-Linked Immunosorbent Assay
PCA	Principal Component Analysis
HDSS	Health and Demographic Surveillance System
ANC	Antenatal Care
RDT	Rapid Diagnostic Test
OD	Optical Density
CRUN	Clinical Research Unit of Nanoro
